# Early-life famine exposure and subsequent risk of chronic disease comorbidity in later adulthood: the role of social activities

**DOI:** 10.3389/fnut.2025.1532731

**Published:** 2025-04-08

**Authors:** Rui Zhao, Qi Zheng, Le-qin Chen, Qiang Feng

**Affiliations:** ^1^China Institute of Sport Science, Beijing, China; ^2^School of Physical Education, Shanxi Normal University, Taiyuan, China

**Keywords:** famine, Chinese Great Famine, chronic disease comorbidity, chronic disease, social activities

## Abstract

**Background:**

With the global population aging, the burden of health issues has shifted from infectious diseases to chronic diseases. Research indicates a significant link between exposure to famine in early life and chronic diseases. However, evidence regarding the relationships among early-life famine exposure, social activities, and chronic disease comorbidities is lacking.

**Objectives:**

We aimed to systematically assess how early-life famine exposure influences the risk of chronic disease comorbidities in later adulthood and how social activities modulate this risk.

**Methods:**

In this nationwide study, we utilized data from the fourth wave of the CHARLS conducted in 2018. We included 6,641 participants and categorized them into five groups based on birth dates. We used the LCA model to reclassify the 14 chronic diseases from the CHARLS survey as the main outcome indicators. We employed stepwise logistic regression to examine the link between early-life famine exposure and the subsequent risk of chronic disease comorbidity in adulthood, presenting the findings as ORs and 95% CIs. We conducted subgroup analyses according to baseline characteristics to examine the robustness and potential differences in outcomes for chronic disease comorbidity. We evaluated the interaction between famine exposure and social engagement on both additive and multiplicative scales using generalized linear models (GLM).

**Results:**

The prevalence of chronic disease comorbidity patterns between 1.3% (cancer-related disease) and 19.9% (cardiovascular disease comorbidity). Those who experience famine in early life face a heightened risk of cardiovascular disease comorbidity in late adulthood, with an OR (95% CI) of 1.42 (1.12 to 1.80), urinary system disease with an OR (95% CI) of 1.87 (1.05 to 3.34), and multimorbidity with an OR (95% CI) of 1.39 (1.07 to 1.79) compared to those who did not experience such conditions. Participating in social activities can lower the risk of metabolic disease comorbidities in late adulthood for infants who experienced famine, with an (OR [95% CI] of 0.64 [0.43 to 0.97]). There was an interactive effect on the additive (OR [95% CI] of −0.42 [−2.52 to −0.32], *P* < 0.01) and multiplicative (*P* = 0.001) effect between infants with famine exposure and social activity.

**Conclusion:**

Experiencing famine in early life is associated with a heightened risk of chronic disease comorbidities in later adulthood, a relationship modulated by participation in social activities. Social activities and early life exposure to famine have an interactive effect on chronic disease comorbidities in later adulthood.

## Introduction

As the global population continues to age, the proportion of China’s population aged 65 and above will significantly increase from currently 15.4% to 26% in the year 2050 (1). This demographic shift will lead to a change in health concerns, moving the focus from infectious diseases to chronic diseases (2). In fact, chronic diseases accounted for 88.5% of all fatalities in China, with cardiovascular diseases, cancers, and chronic respiratory diseases representing 80.7% of these deaths (3). Notably, multiple chronic diseases often coexist among the elderly population and show significant clustering characteristics (4). Thus, identifying and intervening modifiable risk factors, such as social engagement and unhealthy dietary practices, is crucial. These interventions not only decrease the likelihood of chronic diseases but also diminish the chances of serious complications among older adults with chronic conditions (5, 6). Moreover, recent studies have demonstrated a significant association between famine exposure and an increased risk of chronic diseases in later adulthood (7, 8). However, it remains unclear whether this association also increases the risk of chronic disease comorbidity in later adulthood, and whether identifying and intervening in modifiable risk factors could mitigate this risk.

In recent years, several studies based on large-scale epidemiological surveys have explored the link between famine and the long-term risk of developing chronic diseases. For instance, evidence from the famine in Ukraine (1932–1933) in 16 Soviet republics suggests that fetal exposure to the famine is linked to a heightened risk of developing diabetes in adulthood (9). Similarly, studies conducted in China have revealed an association between famine exposure during fetal development and long-term arthritis. Additionally, the research found that famine exposure during infancy and school age is associated with chronic diseases such as dyslipidemia and depressive symptoms (10–12). However, these studies have not fully considered the potential impact of comorbid chronic diseases and the issue of non-overlapping birth dates on chronic diseases (13–15). Therefore, it is necessary to control for age and further examine the long-term impact of famine exposure at different stages of life on chronic disease comorbidity, while actively exploring potential factors that could effectively modulate and improve chronic disease risk (16).

Social activities, as a vital component of social relationships, are regarded as effective health intervention measures. In the United States, a study involving 5,381 participants from diverse ethnic backgrounds with atherosclerosis demonstrated the positive role of social support in preventing chronic diseases by evaluating the relationship between social participation and chronic conditions (17). Additionally, a prospective study conducted in China found that active involvement in social activities protects against cognitive decline (6). However, there is currently no evidence to suggest that social activity has an impact on the connection between early-life famine exposure and the subsequent risk of chronic disease comorbidities in adulthood.

In this research, we aimed to systematically evaluate the connection between early-life famine exposure and the risk of chronic disease comorbidities in later adulthood, as well as the moderating effect of social activities on this risk. We hypothesize that: (1) Exposure to famine in early life is linked to a higher likelihood of chronic disease comorbidities in adulthood; (2) Engagement in social activities will positively impact the relationship between early famine exposure and chronic disease comorbidity in later adulthood; and (3) Assessing famine exposure and increasing social engagement positively combine to reduce chronic disease comorbidities.

## Materials and methods

### Study design and participants

**FIGURE 1:**
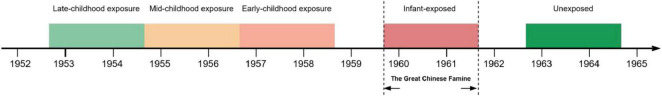
The classification of famine exposure groups based on birth dates.

Data were sourced from Wave 4 of the CHARLS conducted in 2018, which is a national survey comprising 19,816 participants aged 45 and above from 28 of the 31 provinces in mainland China, excluding Tibet, Ningxia, and Hainan (18). Participants were selected using a multistage stratified random sampling approach (19). Drawing from our past research on famine in China, we incorporated a total of 6,641 participants, categorized into five groups based on birth dates (Figure 1): The group exposed during infancy consisted of 905 individuals born between October 1, 1959, and September 30, 1961; the early-childhood exposure group included 1,260 individuals born from October 1, 1956, to September 30, 1958; the mid-childhood exposure group had 1,408 individuals born between October 1, 1954, and September 30, 1956; the late-childhood exposure group involved 1,360 individuals born from October 1, 1952, to September 30, 1954; Finally, the non-exposed group included 1,708 individuals born between October 1, 1962, and September 30, 196. The data used in this study were obtained from the publicly available (CHARLS) database, which has received approval from the Ethics Review Committee of Peking University (No. IRB00001052-11015).

### Assessment of famine exposure and severity

The Great Famine, which occurred between 1959 and 1962, had widespread effects across mainland China; however, the extent of its impact varied across provinces because of differences in climate, population density, and regional food policies. Consequently, we quantified the severity of famine by assessing the excess death rate (EDR) for each province (20). Initially, we determined the province of each individual using the community ID variable from the PSU data (21). We matched this information with the population data from the China Statistical Yearbook to calculate the EDR. Second, we established a cutoff point of 50% EDR (22). Provinces with an EDR below this value were classified as experiencing milder famine conditions, whereas those with an EDR equal to or exceeding this level were considered to have undergone severe famine exposure.

### Outcomes

The outcome variables consisted of multimorbidity and chronic disease comorbidity. Multimorbidity is typically defined as at least two of the 14 chronic conditions in a single individual (23). With chronic disease comorbidity, we used 14 chronic diseases from the 2018 CHARLS survey as the classification object. First, we used the Latent Class Analysis (LCA) model to explore classifications ranging from 1 to 6, comprehensively evaluating and selecting the best model. Then, we calculated the conditional probability and category probability based on this model. Finally, we named each group of features. The specific chronic disease comorbidity pattern categories include cardiovascular disease comorbidity (hypertension, heart disease, stroke), metabolic disease comorbidity (dyslipidemia, diabetes), musculoskeletal disease comorbidity (arthritis), digestive system disease comorbidity (liver disease, stomach disease), respiratory system disease comorbidity (chronic lung disease, asthma), nervous system disease comorbidity (emotional problems, memory-related diseases), urinary system disease (kidney disease), and cancer-related diseases (24).

Based on existing studies, we evaluated the following covariates: gender, education level, marital status, residential area, smoking status, drinking habits, physical activity, social activity, and others. The detailed information is recorded in the Supplementary Table 1.

### Assessment of physical activity

The CHARLS database classifies physical activity (PA) into three levels: vigorous, moderate, and low intensity. Using respondents’ reports on the type and duration of PA (0 min, 10–29 min, 30–119 min, 120–239 min, ≥ 240 min), we calculated the average daily duration using the midpoints for each time interval (25). The exercise volume for various activities was determined using metabolic equivalents (METs) based on the International Physical Activity Questionnaire (IPAQ) guidelines: walking was assigned a MET value of 3.3, moderate activities 4.0, and vigorous activities 8.0 (26). The weekly volume of PA was calculated using the formula: Weekly PA (METs/week) = MET value for the activity * minutes per day * days per week. Finally, weekly PA levels were categorized into three groups: low (< 600 METs/week), moderate (600–3,000 METs/week), and high (> 3,000 METs/week) (27) (Supplementary Methods).

### Statistical analysis

Categorical variables, such as gender, region, and education, were summarized with counts and percentages, whereas continuous variables, like age, were presented as means with standard deviations. We measure the severity of famine using a 50% EDR (less severely < 50% EDR, severely ≥ 50% EDR).

First, we checked for multicollinearity among variables using the Variance Inflation Factor (VIF). Subsequently, we employed stepwise logistic regression to investigate the relationship between early-life famine exposure and the risk of chronic disease comorbidity in adulthood, along with the impact of social activities in this context. We constructed stepwise logistic regression models: Model 1 was unadjusted; Model 2 included controls for factors such as gender, residence, education, marital status, and famine severity; and Model 3 added further adjustments for smoking status, drinking habits, physical activity levels, and social activity.

We separate the interactions between famine groups (non-exposed, infant-exposed, early-childhood exposure, mid-childhood exposure, and late-childhood exposure) and social activities (inactive, active). The non-exposed and inactive were used as the reference group. GLM was used on the additive and multiplicative scales to analyze an adjusted model. The *P*-value reflects the interaction on the multiplicative scale; relative excess risk due to interaction (RERI), attributable proportion of interaction (APAB), Synergy Index (S) represents the interaction effect at the additive scale, when RERI > 0, APAB > 0, and S > 1, or when RERI < 0, APAB < 0, and S < 1 all hold simultaneously, it is considered that there is an additive interactive effect (28, 29).

We conducted stratified analyses based on gender (male, female), residence (rural, urban), and social activities to examine the robustness and potential differences in outcomes for chronic disease comorbidity across different moderating factors. To account for the influence of per capita income on chronic diseases, we conducted sensitivity analyses by incorporating the per capita income variable into the original multivariate adjustment model. Additionally, we substituted the categorical physical activity variable with a continuous measure of physical activity to evaluate chronic disease comorbidity. Data analysis was conducted using R version 4.4.0. The findings are reported as ORs with corresponding 95% CIs, and *p*-values below 0.05 were deemed statistically significant.

## Results

The study included 6,641 participants, of whom 3,410 (51.3%) were female and 3,231 (48.7%) were male. The prevalence of individual chronic diseases varied between 1.1% for psychiatric conditions and 11.2% for hypertension (Supplementary Table 2). Overall, 27.5% of the participants reported being affected by at least one chronic disease, while 16.51% had experienced two or more chronic conditions (Supplementary Figure 1). The prevalence of chronic disease comorbidity patterns ranged from 1.3% (cancer-related disease) to 19.9% (cardiovascular disease comorbidity) (Supplementary Figure 2).

Our study indicates that individuals who faced famine in their early life, especially during childhood, typically demonstrated lower levels of educational attainment and reduced physical activity (PA) compared to those who did not experience such circumstances (Table 1).

**TABLE 1:** The characteristics of the study population.

Characteristic	Overall	Non-exposed	Infant-exposed	early-childhood exposure	mid-childhood exposure	late-childhood exposure
Sample size, *n* (%)	6,641	1,708 (25.7)	905 (13.6)	1,260 (18.9)	1,408 (21.2)	1,360 (20.4)
**Gender, *n* (%)**						
Male	3,231 (48.7)	827 (48.4)	423 (46.7)	645 (51.2)	683 (48.5)	653 (48.0)
Female	3,410 (51.3)	881 (51.6)	482 (53.3)	615 (48.8)	725 (51.5)	707 (52.0)
Age	60.1 ± 3.8	54.8 ± 0.7	57.8 ± 0.7	60.8 ± 0.7	62.8 ± 0.7	64.7 ± 0.7
**Regions, *n* (%)**						
Rural	4,928 (74.2)	1,238 (72.5)	655 (72.4)	931 (73.9)	1,068 (75.9)	1,036 (76.2)
Urban	1,664 (25.1)	455 (26.6)	244 (27.0)	321 (25.5)	330 (23.4)	314 (23.1)
**Education level, *n* (%)**						
Primary school or lower	3,863 (58.2)	796 (46.6)	415 (45.9)	706 (56.0)	920 (65.3)	920 (65.3)
Junior high school	1,689 (25.4)	611 (35.8)	242 (26.7)	317 (25.2)	293 (20.8)	293 (20.8)
Senior high school	992 (14.9)	260 (15.2)	234 (25.9)	222 (17.6)	178 (12.6)	178 (12.6)
University and above	97 (1.5)	41 (2.4)	14 (1.5)	15 (1.2)	17 (1.2)	17 (1.2)
**Marital status (%)**						
Married/cohabiting	5,566 (83.8)	1,453 (85.1)	752 (83.1)	1,064 (84.4)	1,172 (83.2)	1,125 (82.7)
Widowed/single/ divorced/separated	1,075 (16.2)	255 (14.9)	153 (16.9)	196 (15.6)	236 (16.8)	235 (17.3)
Per capita income, CNY	6,327.0 ± 13,022.6	6,175.5 ± 12,742.1	5,607.3 ± 10,633.0	7,159.1 ± 14,668.3	6,136.8 ± 12,632.3	6,395.3 ± 13,497.0
**PA, *n* (%)**						
Low	3,007 (45.3)	707 (41.4)	426 (47.1)	576 (45.7)	328 (44.6)	670 (49.3)
Middle	3,205 (48.3)	882 (51.6)	415 (45.9)	604 (47.9)	680 (48.3)	624 (45.9)
High	429 (6.5)	119 (7.0)	64 (7.1)	80 (6.3)	100 (7.1)	66 (4.9)
**Social activity, *n* (%)**						
Inactive	3,091 (46.5)	694 (40.6)	422 (46.6)	570 (45.2)	699 (49.6)	706 (51.9)
Active	3,550 (53.5)	1,014 (59.4)	483 (53.4)	690 (54.8)	709 (50.4)	654 (48.1)
Smoking, never, No. (%)	3,704	1,003 (27.1)	526 (14.2)	676 (18.3)	756 (20.4)	743 (20.1)
**Drinking habits, *n* (%)**						
Never drinking	4,263 (64.2)	1,060 (62.1)	571 (63.1)	813 (64.6)	909 (64.6)	910 (67.0)
≤ 1 time/month	501 (7.5)	143 (8.4)	66 (7.3)	94 (7.5)	104 (7.4)	94 (6.9)
> 1 time/month	1,872 (28.2)	504 (29.5)	268 (29.6)	351 (27.9)	394 (28.0)	355 (26.1)
**Famine severity, *n* (%)**						
Less severely	3,771 (56.8)	922 (54.0)	569 (62.9)	734 (58.3)	800 (56.8)	746 (54.9)
Severely	2,870 (43.2)	786 (46.0)	336 (37.1)	526 (41.7)	608 (43.2)	614 (45.1)

Continuous data are reported as the mean (sd), and categorical data are reported as the number and percentage of participants.

Table 2 illustrates the relationship between various stages of famine exposure and the risk of developing chronic disease comorbidities in later life. Research indicates that individuals who experience famine in early life face a heightened risk of comorbid cardiovascular disease, urinary system disease, and multimorbidity. After controlling for confounding factors such as gender, residence, and education, the findings remain robust: cardiovascular disease comorbidity OR (95% CI) of 1.42 (1.12 to 1.80), urinary system disease OR (95% CI) of 1.87 (1.05 to 3.34), and multimorbidity OR (95% CI) of 1.39 (1.07 to 1.79). To more accurately assess the impact of famine exposure on the risk of chronic disease comorbidity, we conducted a sensitivity analysis on age. The analysis results indicated that the research findings remained largely consistent before and after adjusting for age differences (Supplementary Table 3 and Supplementary Figure 3).

**TABLE 2:** Association between famine exposure and subsequent chronic disease comorbidity in adulthood.

	Model 1 : Crude model OR (95% CI)					Model 2: Adjusted model OR (95% CI)				
	**Unexposed**	**Infant-exposed**	**Early-childhood exposure**	**Mid-childhood exposure**	**Late-childhood exposure**	**Unexposed**	**Infant-exposed**	**Early-childhood exposure**	**Mid-childhood exposure**	**Late-childhood exposure**
Cardiovascular disease comorbidity	Ref	1.10 (0.89–1.36)	**1.24 (1.03–1.50)**	**1.27 (1.06–1.52)**	**1.44 (1.21–1.73)**	Ref	1.12 (0.28–0.68)	1.24 (0.97–1.58)	1.21 (0.95–1.54)	**1.42 (1.12–1.80)**
Metabolic disease comorbidity	Ref	0.90 (0.71–1.13)	1.10 (0.89–1.35)	0.94 (0.77–1.15)	0.99 (0.81–1.22)	Ref	1.00 (0.75–1.34)	1.21 (0.93–1.57)	1.03 (0.79–1.33)	1.12 (0.86–1.45)
Musculoskeletal disease comorbidity	Ref	1.17 (0.87–1.58)	0.94 (0.71–1.26)	0.95 (0.72–1.26)	1.11 (0.84–1.46)	Ref	1.32 (0.91–1.91)	0.95 (0.65–1.37)	1.02 (0.72–1.44)	1.04 (0.72–1.50)
Digestive system disease comorbidity	Ref	1.03 (0.78–1.35)	0.92 (0.72–1.19)	1.00 (0.79–1.28)	1.10 (0.87–1.40)	Ref	1.15 (0.82–1.60)	1.03 (0.75–1.41)	0.83 (0.60–1.14)	1.20 (0.88–1.63)
Respiratory system disease comorbidity	Ref	1.05 (0.74–1.48)	1.10 (0.8–1.50)	1.17 (0.87–1.58)	**1.50 (1.12–1.99)**	Ref	1.25 (0.81–1.93)	0.85 (0.55–1.31)	1.00 (0.67–1.50)	1.20 (0.81–1.77)
Nervous system disease comorbidity	Ref	**1.77 (1.02–3.07)**	**1.67 (1.00–2.80)**	**1.96 (1.21–3.19)**	**2.43 (1.51–3.89)**	Ref	1.32 (0.69–2.51)	1.46 (0.82–2.61)	1.16 (0.64–2.10)	1.59 (0.91–2.77)
Urinary system disease	Ref	**1.80 (1.16–2.81)**	1.26 (0.81–1.97)	**1.59 (1.05–2.40)**	**1.54 (1.02–2.34)**	Ref	**1.87 (1.05–3.34)**	1.22 (0.67–2.22)	1.54 (0.88–2.68)	1.48 (0.84–2.60)
Cancer-related disease	Ref	**2.19 (1.06–4.50)**	1.46 (0.70–3.04)	1.92 (0.98–3.77)	1.80 (0.91–3.58)	Ref	2.09 (0.87–4.99)	1.56 (0.65–3.72)	1.69 (0.74–3.88)	1.56 (0.67–3.65)
Multimorbidity	Ref	1.07 (0.85–1.34)	**1.30 (1.07–1.59)**	**1.29 (1.06–1.56)**	**1.39 (1.15–1.69)**	Ref	1.31 (0.99–1.74)	**1.40 (1.08–1.80)**	1.26 (0.98–1.62)	**1.39 (1.07–1.79)**

Ref, reference. Model 1: unadjusted. Model 2 adjusted for gender, residence, education, marital status, severity, smoking status, drinking habits, PA, and social activity. The bold values indicate statistically significant.

We treated PA as a continuous variable and incorporated a per capita income metric to assess the robustness of the findings. Sensitivity analyses revealed that these results remained broadly consistent (Supplementary Tables 4, 5).

**FIGURE 2:**
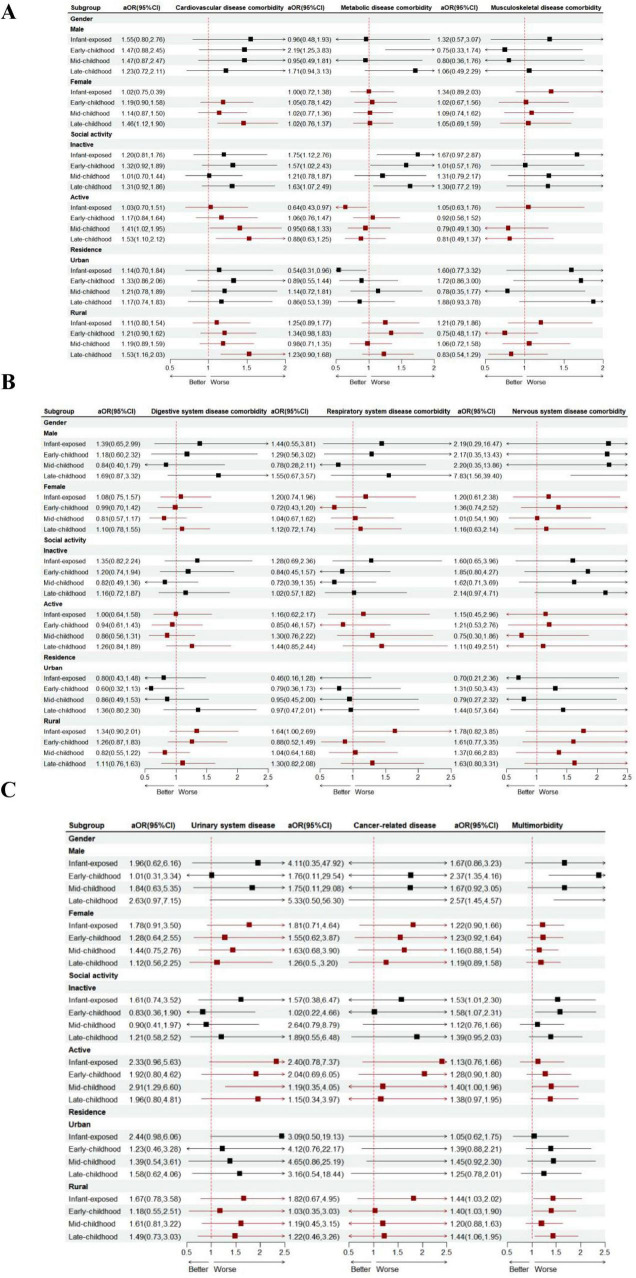
Subgroup analyses of the association between famine exposure and the risk of chronic disease comorbidity **(A,B,C)** are presented. Note: The dots represent the OR comparing unfavorable versus favorable groups of research factors, while the horizontal lines indicate the 95% CIs. The model was adjusted for gender, residence, education, marital status, severity, smoking status, drinking habits, PA, and social activity. The strata variable was excluded from the model when it was used for stratification on its own.

The impact of famine exposure on chronic disease comorbidity in later adulthood varies across subgroups. Notably, among women, the association with cardiovascular disease comorbidity is most pronounced for those exposed to famine in late childhood. Similarly, in men, famine exposure is most strongly linked to multimorbidity and nervous system disease comorbidity when it occurs during late childhood. For rural elderly individuals, the link between famine exposure and cardiovascular disease comorbidity, as well as multimorbidity, is especially pronounced among those who endured famine in late childhood. In contrast, among urban elderly individuals, the risk of metabolic disease comorbidity due to famine exposure is lower in those who were exposed during infancy and early childhood compared to other groups; Participation in social activities can significantly reduce the risk of metabolic disease comorbidity associated with famine exposure, with the most pronounced benefits observed in individuals who experienced famine during infancy. However, our study also reveals that the role of social activity is not universally positive. In populations that experienced famine during childhood, engaging in social activities may increase the risk of cardiovascular disease comorbidity (Figure 2).

An overall analysis of the interaction between famine and social activities reveals a multiplicative effect (*P* = 0.001) and additive effect OR (95% CI) of −1.42 (−2.52 to −0.32) of famine exposure during infancy on participation in social activities is significant (Table 3 and Supplementary Tables 6–8).

**TABLE 3:** The interactions between Infant-exposed group and social activities on metabolic disease comorbidity.

	Inactive		Active		OR (95%CI) for metabolic disease comorbidity within strata of famine exposure
	***N***	**OR (95%CI)**	***N***	**OR (95%CI)**	
Unexposed group	694	1.00 (Ref)	1,014	2.08 (1.41,3.06)	2.08 (1.41,3.06), *P* < 0.01**
Infant-exposed group	422	1.76 (1.12,2.76)	483	0.64 (0.43,0.97)	0.81 (0.49,1.33), *P* = 0.40
ORs (95% CI) for metabolic disease comorbidity within strata of social activity	1.86 (1.16,2.96), *P* < 0.01**		0.66 (0.44,0.10), *P* < 0.05*		–
RERI^#^	−1.42 (−2.52, −0.32),RERI < 0				
APAB	−1.11 (−2.05,−0.17),APAB < 0				
S	S = 0.167				
*P*_ interaction_ *	*P* = 0.001				

RERI, relative excess risk due to interaction; APAB, attributable proportion of interaction; S, Synergy Index; Ref, reference.

^#^Measure of interaction on the additive scale.

*Measure of interaction on the multiplicative scale.

**The effect of inactive on metabolic diseases was statistically significant. ORs are adjusted for gender, residence, education, marital status, severity, smoking status, drinking habits, PA.

## Discussion

In this national sample study, we examined the association between early-life famine and the development of chronic disease comorbidities, along with the influence of social activities. We observed that individuals who experienced famine early in life are highly susceptible to chronic disease comorbidity, particularly cardiovascular disease comorbidity and urinary system disease. Additionally, the risk of chronic disease comorbidity varies by gender, residential area, and social activity. Notably, we found that participation in social activities is associated with chronic disease comorbidities and that social activities and famine exposure interact with chronic disease comorbidity.

Due to declining physical function and weakened immunity, elderly adults are prone to developing multiple chronic diseases and complex comorbidity patterns (24, 30). In China, the prevalence of chronic disease comorbidity among adults reaches up to 50% and may continue to grow at a rate of 1% annually, placing a significant strain on families and healthcare systems (23, 31). Our study shows that experiencing famine early in life increases the risk of comorbidities in adulthood, primarily observed as a pattern of cardiovascular disease comorbidity and urinary system diseases, which accords with the previous research findings (15). The possible mechanism behind this result is that famine leads to prolonged malnutrition, and the kidneys adapt to energy deficiency by regulating nitrogen balance, electrolytes, and acid-base balance. However, the sustained metabolic burden and nitrogen retention processes, such as glutamine metabolism and ketone body utilization, increase the kidney’s burden, especially in handling ammonia and urine excretion. Additionally, kidney cells mobilize fatty acids for energy through lipid metabolism and autophagy, but excessive metabolic stress and defects in autophagy can impair cellular function. In prolonged states of hunger, impaired fatty acid mobilization and autophagic degradation may lead to insufficient energy supply to the kidneys, electrolyte imbalances, cell death, and kidney damage, thereby increasing the risk of kidney disease (32, 33). Compared to current chronic disease comorbidity research, most studies primarily focus on analyzing the long-term impact of famine on a single chronic disease without adequately considering the role of comorbidities. The results of this study indicate that, in the analysis of metabolic disease comorbidities, the morbidity rate in the famine-exposed group did not show a significant increase compared to the pre-famine and post-famine control groups. This finding is consistent with the results of a systematic review that included 23 original studies. However, some review studies have reached different conclusions, reporting that the risk of developing type 2 diabetes in the famine-exposed population was 1.4 times higher than that in the control group (34–36). Further analysis suggests that this difference may primarily stem from methodological limitations, particularly the absence of key information (such as age balance), which could impact the reliability of the study’s conclusions (37). For example, Chihua Li et al. after appropriately controlling for age,

compared the chronic disease outcomes between the exposed and unexposed groups and found that the impact of famine on chronic diseases was limited (38). It is worth noting that the heterogeneity in current research findings on famine and chronic diseases may also be attributed to inconsistent methods for assessing the severity of famine across different regions, familial socioeconomic status, and variations in study design and outcome measurements (37, 39). These methodological differences could affect the reliability of the study conclusions. Therefore, future studies should adopt more refined analytical methods, account for potential confounding factors, and standardize assessment criteria to more accurately evaluate the impact of famine exposure on long-term health.

The connection between famine exposure and chronic disease comorbidity shows distinct variations across different subgroups. Our findings indicate that early-life famine exposure correlates with a 7.8-fold increase in the risk of nervous system disease comorbidity among men, though no such association is observed in the overall population. The reasons for this variation are not fully understood and may relate to differing health risk factors by gender, such as the higher prevalence of smoking and alcohol consumption among men in China, which are closely linked to chronic diseases (40). Our study also emphasizes the urban-rural disparities in how early-life famine exposure affects chronic disease comorbidity in later adulthood. In rural areas, we observed that those who experienced famine face a greater risk of multimorbidity. Possible explanations for the differences in this correlation include issues during the Great Famine related to the collectivization movement, natural disasters, and human factors that led to reduced food production, compounded by restrictions on rural-to-urban migration that exacerbated the severity of rural famine. Additionally, the faster aging rate and economic and healthcare disadvantages in rural areas compared to urban areas may further increase this risk (41). Exposure to famine during infancy and early childhood is closely associated with the comorbidity of metabolic diseases. Malnutrition, especially under starvation conditions, affects fatty acid mobilization and metabolism through various mechanisms, leading to metabolic disturbances. In a state of hunger, cells acquire fatty acids through autophagy and transfer them from lipid droplets to the mitochondria for oxidative metabolism. The transport and metabolism of fatty acids are finely regulated by cytosolic lipases and mitochondrial fusion. When mitochondrial fusion is impaired, fatty acids cannot be properly metabolized, rebind to lipid droplets, and are transferred to adjacent cells, further exacerbating metabolic disturbances (42, 43). Additionally, an animal study has shown that when protein intake is restricted during pregnancy, offspring are more prone to developing metabolic diseases. This finding suggests that early malnutrition not only directly impairs cellular oxidative metabolism by affecting fatty acid mobilization and metabolism but also alters an individual’s metabolic response to later dietary intake, particularly to high sugar consumption, increasing the risk of developing metabolic diseases in adulthood. This mechanism highlights the long-term impact of early nutritional status on metabolic health and emphasizes the importance of improving maternal and early-life nutrition to reduce the incidence of metabolic diseases (44).

This study reveals that social activities can reduce metabolic risks associated with early-life famine exposure, and there is an interactive effect between social activities and early-life famine exposure. Specifically, actively participating in social activities lowers the risk of metabolic disease comorbidity linked to infant famine exposure by 36%. This finding is consistent with previous results obtained in diabetic populations; a study involving 726 patients with type II diabetes found that social support can enhance blood glucose control (45). Prior studies suggest that increasing social activities may counteract insulin resistance through complex interactions among various organs (46), Similarly, a study of 88,000 adults born before and after the Chinese Great Famine found that, after correcting for age imbalance, fetal exposure to famine could lead to permanent changes in the function and number of pancreatic ββ-cells and the tissue’s sensitivity to insulin (47), thereby indirectly improving metabolic function. Concurrently, nutritional issues, such as famine exposure, may lead to increased insulin resistance in pancreatic beta cells (47–49).

Based on the mechanisms by which social activities influence the relationship between famine and metabolic diseases, we propose that tailored social interventions for populations facing nutritional challenges may be a viable solution for mitigating and alleviating the co-burden of chronic diseases.

Moreover, our study shows that the influence of social activities on chronic disease comorbidity is not always positive. For example, social activities have been associated with a heightened risk of cardiovascular disease comorbidity (50). Additionally, ineffective social engagement correlates with increased rates of depression (51). Therefore, a more thorough investigation is necessary to clarify the role of social activities in the connection between famine and chronic disease comorbidity.

To our knowledge, this study is the first to utilize a nationally representative sample from China, controlling for age differences between comparison groups caused by non-overlapping birth dates, to systematically investigate the association between early-life exposure to famine and the risk of comorbid chronic diseases in adulthood. Second, we employed extensive, nationally representative data and appropriate statistical methods to establish a strong association between famine exposure and chronic disease comorbidities, providing a detailed analysis across various subpopulations. Third, this research is the first to illustrate the interactive effects of social activity and famine exposure on chronic disease comorbidity in older adults.

This study has several limitations. First, the cross-sectional nature of the data may not accurately capture the current multimorbidity status among older adults. Our national cross-sectional surveys cannot infer causality; therefore, longitudinal studies are needed in the future to explore this relationship in greater depth. Second, the absence of income data may bias the observed relationship between early-life famine exposure and subsequent chronic disease comorbidity in adulthood. To address this, we made the best effort to process income data and calculated total physical activity as a continuous variable for sensitivity analysis. Third, data collection through questionnaires combined with face-to-face computer-assisted personal interviews may introduce recall bias for certain measures, such as physical activity. However, the large-scale national survey minimized errors as much as possible. EDR, as an indicator of famine severity, primarily relies on mortality data. It may be influenced by factors such as incomplete reporting, statistical delays, and inconsistencies in data collection methods, which could lead to an underestimation or overestimation of the actual mortality burden. Forth, the exclusion of migration data from our sample could potentially give rise to bias; however, previous research suggests that provincial migration was restricted by policy, preventing individuals from moving across regions significantly affected by the famine (52, 53). Last, although this study provides valuable insights into the impact of famine exposure on the burden of chronic diseases, there are still certain limitations in the in-depth exploration of its public health significance and cost evaluation (54, 55). Future research should further deepen the understanding of the relationship between famine and chronic diseases, particularly by analyzing their potential impact on public health systems and socio-economic structures. In addition, more emphasis should be placed on utilizing existing research data and cost-effectiveness analysis methods to guide the formulation of public health policies, thereby effectively reducing the long-term health costs associated with famine exposure.

In conclusion, experiencing famine in early life is associated with a heightened risk of chronic disease comorbidities in later adulthood, a relationship modulated by participation in social activities. Social activities and early life exposure to famine have an interactive effect on chronic disease comorbidities in later adulthood. Future research should explore specific social activity strategies that can further enhance health outcomes.

## Data Availability

The datasets presented in this study can be found in online repositories. The names of the repository/repositories and accession number(s) can be found in this article/Supplementary material.
