# Supplementary material for: Early-life famine exposure and subsequent risk of chronic disease comorbidity in later adulthood: the role of social activities
Source: Front Nutr. 2025 Apr 8;12:1532731. doi: 10.3389/fnut.2025.1532731 (PMC12011593; doi:10.3389/fnut.2025.1532731)
Supplement: Supplementary file 1 [file Table_1.docx]

**Supplementary appendix**

Early-life Famine Exposure and Subsequent Risk of Chronic Disease Comorbidity in Later Adulthood: The Role of Social Activities

**Supplementary appendix**

**[eTable 1](#_Toc17149)** [Harmonized definitions for key covariates in the study 2](#_Toc17149)

**[eMethods](#_Toc29244)** [MET values and physical activity intensity categories 3](#_Toc29244)

**[eTable 2](#_Toc12836)** [Questionnaire items and prevalence of chronic diseases in CHARLS 4](#_Toc12836)

**[eTable 3](#_Toc17905)** [Effect estimates on chronic disease comorbidity comparing famine births with controls (post-famine births) 5](#_Toc17905)

**[eTable 4](#_Toc6833)** [Association between famine and subsequent chronic disease comorbidity in adulthood: using PA continuous variable 6](#_Toc6833)

**[eTable 5](#_Toc3051)** [Association between famine and subsequent chronic disease comorbidity in adulthood: additionally adjusted for per capita income 7](#_Toc3051)

**[eTable 6](#_Toc18942)** [The interactions between early-childhood group and social activities on metabolic disease comorbidity 8](#_Toc18942)

**[eTable 7](#_Toc31217)** [The interactions between mid-childhood group and social activities on metabolic disease comorbidity 9](#_Toc31217)

**[eTable 8](#_Toc3395)** [The interactions between late-childhood group and social activities on metabolic disease comorbidity 10](#_Toc3395)

**[eFigure 1](#_Toc1213)** [The quantity and prevalence of chronic disease types 11](#_Toc1213)

**[eFigure 2](#_Toc31018)** [The prevalence of chronic disease comorbidity patterns 11](#_Toc31018)

**[eFigure 3](#_Toc32199)** [Sensitivity Analysis on the Risk of Comorbidity between Famine Exposure and Chronic Diseases: using Age-Period-Cohort Analysis 12](#_Toc32199)

**[Ethics approval](#_Toc13048)** [13](#_Toc13048)

**[Funding](#_Toc20539)** [13](#_Toc20539)

**[References](#_Toc28703)** [14](#_Toc28703)

# **eTable 1** Harmonized definitions for key covariates in the study

| Variables | Values | |
| --- | --- | --- |
| Education | Primary school or lower | No formal education (illiterate)/Did not finish primary schoo/Sishu/home school/Elementary school/ |
|  | Junior high school | Middle school |
|  | Senior high school | High school/Vocational school |
|  | University and above | Two-/Three-Year College/Associate degree/Four-Year College/Bachelor’s degree/Maste’s degree/Doctoral degree/Ph.D. |
| Marital | Married or partnered | Married and living together with |
|  | Single | Widowed/Single/Divorced/Separated |
| Social activity | Inactive | Respondents did not participate any social activities or groups in last month. |
|  | Active | Respondents participated at least one social activities or groups in last month. |
| Cigarette smoking | No | Never |
|  | Yes | Previous, and current |
| Alcohol drinking | No | Never |
|  | Yes | ≤1 time/month |
|  |  | >1 time/month |
| Famine severity | Less severely | The excess death rate is less than 50%. |
|  | Severely | The excess death rate is greater than 50%. |

# **eMethods** MET values and physical activity intensity categories

According to the CHARLS questionnaire, the duration of daily physical activity types is divided into 5 categories (0 min, 10~29 min, 30~119 min, 120~239 min, ≥240 min), and the median is used to calculate the exercise time for high, moderate, and low-intensity physical activities. The weekly duration of physical activity = number of days performing each type of physical activity × duration of each type per day^1^.

The amount of high-intensity physical activity is quantified using the Metabolic Equivalent (MET), which is a measure of the intensity of physical activity. According to the International Physical Activity Questionnaire (IPAQ) assessment criteria, walking has a MET value of 3.3, moderate-intensity activity has a MET value of 4.0, and high-intensity activity has a MET value of 8.0^2^.

The score of physical activity is calculated by the formula: Physical activity score = 8.0 × total duration of high-intensity activity per week + 4.0 × total duration of moderate-intensity activity per week + 3.3 × total duration of walking per week. The weekly level of physical activity is categorized into low-intensity physical activity (< 600 METs/week), moderate-intensity physical activity (600~3,000 METs/week), and high-intensity physical activity (> 3,000 METs/week)^3^.

# **eTable 2** Questionnaire items and prevalence of chronic diseases in CHARLS

| Types of  Chronic Diseases | Questionnaire Items（codes：DA007） | Prevalence (%) |
| --- | --- | --- |
| Hypertension | Have you been diagnosed with Hypertension by a doctor? | 11.2 |
| Dyslipidemia | Have you been diagnosed with Dyslipidemia (elevation of low density lipoprotein, triglycerides (TGs),and total cholesterol, or a low high density lipoprotein level) by a doctor? | 10.1 |
| Diabetes | Have you been diagnosed with Diabetes or high blood sugar by a doctor? | 5.5 |
| Cancer | Have you been diagnosed with Cancer or malignant tumor (excluding minor skin cancers) by a doctor? | 1.3 |
| Chronic lung disease | Have you been diagnosed with Chronic lung diseases, such as chronic bronchitis , emphysema ( excluding tumors, or cancer) by a doctor? | 5.0 |
| Liver disease | Have you been diagnosed with Liver disease (except fatty liver, tumors, and cancer) by a doctor? | 3.2 |
| Heart disease | Have you been diagnosed with Heart attack, coronary heart disease, angina, congestive heart failure, or other heart problems by a doctor? | 6.4 |
| Stroke | Have you been diagnosed with Stroke by a doctor? | 4.9 |
| Kidney disease | Have you been diagnosed with Kidney disease (except for tumor or cancer) by a doctor? | 3.3 |
| Digestive disease | Have you been diagnosed with Stomach or other digestive diseases (except for tumor or cancer) by a doctor? | 6.9 |
| Psychiatric disease | Have you been diagnosed with Emotional, nervous, or psychiatric problems by a doctor? | 1.1 |
| Memory-related disease | Have you been diagnosed with Emotional, nervous, or psychiatric problems by a doctor? | 1.7 |
| Arthritis | Have you been diagnosed with Arthritis or rheumatism by a doctor? | 7.3 |
| Asthma | Have you been diagnosed with Asthma by a doctor? | 1.9 |

# **eTable 3** Effect estimates on chronic disease comorbidity comparing famine births with controls (post-famine births)

|  | **Model 1 : Crude model OR (95% CI)** | | **Model 2 : Adjusted model OR (95% CI)** | |
| --- | --- | --- | --- | --- |
|  | **Group 0** | **Group 1** | **Group 0** | **Group 1** |
| Cardiovascular  disease comorbidity | Ref | 1.00(0.83-1.21) | Ref | 1.00(0.78-1.28) |
| Metabolic  disease comorbidity | Ref | 0.86(0.69-1.07) | Ref | 0.94(0.72-1.24) |
| Musculoskeletal  disease comorbidity | Ref | 1.20(0.91-1.58) | Ref | 1.36(0.96-1.91) |
| Digestive system  disease comorbidity | Ref | 1.06(0.83-1.37) | Ref | 1.12(0.82-1.53) |
| Respiratory system disease comorbidity | Ref | 1.00(0.73-1.38) | Ref | 1.31(0.88-1.97) |
| Nervous system  disease comorbidity | Ref | 1.38(0.86-2.21) | Ref | 1.10(0.62-1.94) |
| Urinary system disease | Ref | **1.63(1.10-2.40)** | Ref | **1.70(1.02-2.84)** |
| Cancer-related disease | Ref | 1.83(0.99-3.39) | Ref | 1.81(0.86-3.84) |

Note: Group0, controls (post-famine births); Group1, Infant-exposed; Ref, reference. Model 1: unadjusted. Model 2 adjusted for gender, residence, education, marital status, severity, smoking status, drinking habits, PA, and social activity. The bold values indicate statistically significant

# **eTable 4** Association between famine and subsequent chronic disease comorbidity in adulthood: using PA continuous variable

|  | Model : Adjusted model OR (95% CI) | | | | |
| --- | --- | --- | --- | --- | --- |
|  | Group 0 | Group 1 | Group 2 | Group 3 | Group 4 |
| Cardiovascular  disease comorbidity | Ref | 1.12(0.86-1.47) | 1.24(0.97-1.58) | 1.21(0.95-1.54) | **1.42(1.12-1.81)** |
| Metabolic  disease comorbidity | Ref | 1.01(0.75-1.35) | 1.21(0.94-1.57) | 1.03(0.79-1.34) | 1.11(0.86-1.44) |
| Musculoskeletal  disease comorbidity | Ref | 1.32(0.92-1.91) | 0.95(0.65-1.37) | 1.01(0.71-1.44) | 1.04(0.73-1.50) |
| Digestive system  disease comorbidity | Ref | 1.14(0.82-1.60) | 1.03(0.75-1.41) | 0.83(0.60-1.14) | 1.19(0.87-1.61) |
| Respiratory system disease comorbidity | Ref | 1.25(0.81-1.93) | 0.85(0.55-1.31) | 1.00(0.67-1.50) | 1.21(0.82-1.78) |
| Nervous system  disease comorbidity | Ref | 1.33(0.70-2.54) | 1.47(0.82-2.62) | 1.17(0.64-2.11) | 1.60(0.92-2.80) |
| Urinary system disease | Ref | **1.86(1.04-3.32)** | 1.22(0.67-2.21) | 1.54(0.88-2.67) | 1.46(0.83-2.57) |
| Cancer-related  disease | Ref | 2.17(0.91-5.18) | 1.59(0.67-3.78) | 1.74(0.76-3.97) | 1.57(0.67-3.66) |
| Multimorbidity | Ref | 1.32(1.00-1.74) | **1.40(1.08-1.81)** | 1.26(0.98-1.63) | **1.38(1.07-1.78)** |

Note: Group0, Unexposed; Group1, Infant-exposed; Group2, early-childhood exposure; Group3, mid-childhood exposure; Group4, late-childhood exposure; Ref, reference. Model was adjusted for gender, residence, education, marital status, severity, smoking status, drinking habits, PA per week (METs) , and social activity. The bold values indicate statistically significant.

# **eTable 5** Association between famine and subsequent chronic disease comorbidity in adulthood: additionally adjusted for per capita income

|  | Model : Adjusted model OR (95% CI) | | | | |
| --- | --- | --- | --- | --- | --- |
|  | Group 0 | Group 1 | Group 2 | Group 3 | Group 4 |
| Cardiovascular  disease comorbidity | Ref | **2.00(1.12-3.59)** | 1.67(0.96-2.92) | 1.36(0.76-2.43) | **1.82(1.03-3.21)** |
| Metabolic  disease comorbidity | Ref | 1.50(0.80-2.80) | 1.40(0.79-2.47) | 1.28(0.71-2.30) | 1.26(0.70-2.29) |
| Musculoskeletal  disease comorbidity | Ref | 1.56(0.75-3.28) | 0.95(0.44-2.05) | 0.95(0.45-2.03) | 1.68(0.81-3.46) |
| Digestive system  disease comorbidity | Ref | 1.27(0.64-2.51) | 0.93(0.48-1.82) | 0.50(0.23-1.08) | 0.96(0.50-1.88) |
| Respiratory system disease comorbidity | Ref | 1.10(0.46-2.66) | 0.91(0.39-2.12) | 0.74(0.31-1.76) | 1.25(0.58-2.68) |
| Nervous system  disease comorbidity | Ref | 1.77(0.34-9.11) | 3.29(0.85-12.78) | 1.20(0.23-6.19) | **4.00(1.04-15.39)** |
| Urinary system disease | Ref | 0.88(0.21-3.69) | 0.93(0.25-3.51) | 1.26(0.35-4.49) | 0.75(0.18-3.20) |
| Cancer-related  disease | Ref | 2.19(0.60-8.01) | 1.70(0.46-6.24) | 0.80(0.18-3.55) | 0.77(0.17-3.46) |
| Multimorbidity | Ref | 1.44(0.78-2.62) | **1.85(1.09-3.16)** | 0.97(0.54-1.76) | 1.47(0.84-2.57) |

Note: Ref, reference. Model was adjusted for gender, residence, education, marital status, severity, smoking status, drinking habits, PA social activity, and per capita income. The bold values indicate statistically significant.

# **eTable 6** The interactions between early-childhood group and social activities on metabolic disease comorbidity

|  | **Inactive** | | **Active** | | OR (95%CI) for metabolic disease comorbidity within strata of famine exposure |
| --- | --- | --- | --- | --- | --- |
|  | N | OR (95%CI) | N | OR (95%CI) |  |
| Unexposed Group | 694 | 1.00 (Ref) | 1014 | 2.08(1.41,3.06) | 2.08(1.41,3.06),P<0.01** |
| early-childhood Group | 570 | 1.57(1.02,2.43 | 690 | 1.06(0.76,1.47) | 1.48(0.99,2.22),P=0.06 |
| ORs (95% CI) for metabolic disease comorbidity within strata of social activity | 1.61(1.04,2.50),P<0.05^*^ | | 1.05(0.76,1.47),P=0.75 | | - |
| RERI^#^ | -0.43(-1.34,0.48),RERI<0 | | | | |
| APAB | -0.21(-0.66,0.24),APAB<0 | | | | |
| S | S=0.713 | | | | |
| P _interaction_^*^ | P=0.166 | | | | |

Note: RERI, Relative Excess Risk due to Interaction; APAB, Attributable Proportion of Interaction; S, Synergy Index; Ref, reference. ^#^Measure of interaction on the additive scale. ^*^Measure of interaction on the multiplicative scale. ORs are adjusted for gender, residence, education, marital status, severity, smoking status, drinking habits, PA.

# **eTable 7** The interactions between mid-childhood group and social activities on metabolic disease comorbidity

|  | **Inactive** | | **Active** | | OR (95%CI) for metabolic disease comorbidity within strata of famine exposure |
| --- | --- | --- | --- | --- | --- |
|  | N | OR (95%CI) | N | OR (95%CI) |  |
| Unexposed Group | 694 | 1.00 (Ref) | 1014 | 2.08(1.41,3.06) | 2.08(1.41,3.06),P<0.01** |
| mid-childhood Group | 699 | 1.21(0.78,1.87) | 709 | 0.95(0.68,1.33) | 1.55(1.04,2.33),P<0.05^*^ |
| ORs (95% CI) for metabolic disease comorbidity within strata of social activity | 1.20(0.77,1.87),P=0.42 | | 0.95(0.68,1.34),P=0.77 | | - |
| RERI^#^ | -0.14(-0.93, 0.65),RERI<0 | | | | |
| APAB | -0.07(-0.49, 0.34),APAB<0 | | | | |
| S | S=0.865 | | | | |
| P _interaction_^*^ | P=0.609 | | | | |

Note: RERI, Relative Excess Risk due to Interaction; APAB, Attributable Proportion of Interaction; S, Synergy Index; Ref, reference. ^#^Measure of interaction on the additive scale. ^*^Measure of interaction on the multiplicative scale. ORs are adjusted for gender, residence, education, marital status, severity, smoking status, drinking habits, PA.

# **eTable 8** The interactions between late-childhood group and social activities on metabolic disease comorbidity

|  | **Inactive** | | **Active** | | OR (95%CI) for metabolic disease comorbidity within strata of famine exposure |
| --- | --- | --- | --- | --- | --- |
|  | N | OR (95%CI) | N | OR (95%CI) |  |
| Unexposed Group | 694 | 1.00 (Ref) | 1014 | 2.08(1.41,3.06) | 2.08(1.41,3.06),P<0.01** |
| late-childhood Group | 706 | 1.22(0.79,1.88) | 654 | 0.88(0.63,1.25) | 1.11(0.74,1.65),P=0.61 |
| ORs (95% CI) for metabolic disease comorbidity within strata of social activity | 1.68(1.08,2.61),P<0.05^*^ | | 0.87(0.61,1.25),P=0.45 | | - |
| RERI^#^ | -0.60(-1.48, 0.29),RERI<0 | | | | |
| APAB | -0.33(-0.82, 0.17),APAB<0 | | | | |
| S | S=0.583 | | | | |
| P _interaction_^*^ | P=0.085 | | | | |

Note: RERI, Relative Excess Risk due to Interaction; APAB, Attributable Proportion of Interaction; S, Synergy Index; Ref, reference. ^#^Measure of interaction on the additive scale. ^*^Measure of interaction on the multiplicative scale. ORs are adjusted for gender, residence, education, marital status, severity, smoking status, drinking habits, PA.


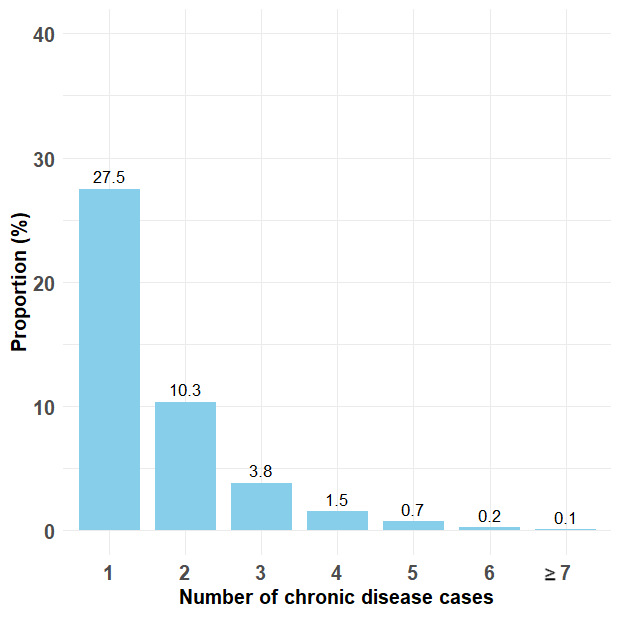


# **e****Figure 1** The quantity and prevalence of chronic disease types


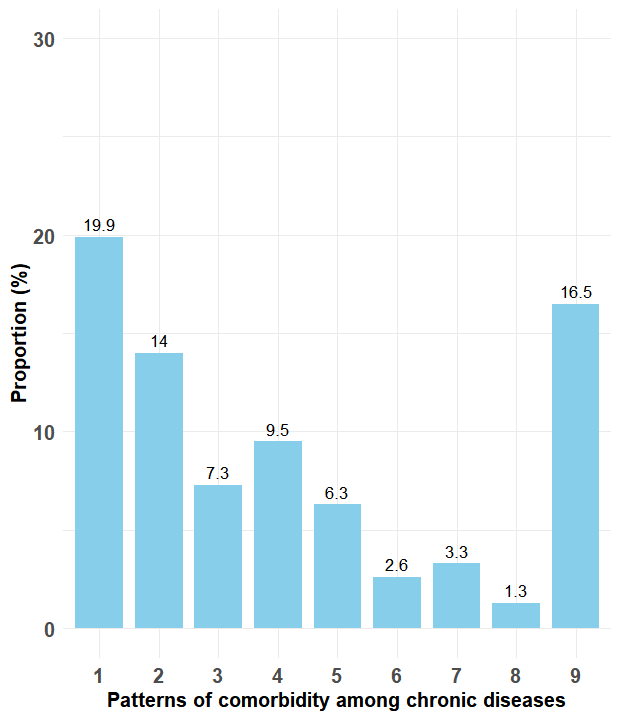


# **eFigure 2** The prevalence of chronic disease comorbidity patterns

Note: 1, Cardiovascular disease comorbidity; 2, Metabolic disease comorbidity; 3, Musculoskeletal disease comorbidity; 4, Digestive system disease comorbidity; 5, Respiratory system disease comorbidity; 6, Nervous system disease comorbidity; 7, Urinary system disease; 8. Cancer-related disease; 9,Multimorbidity

| 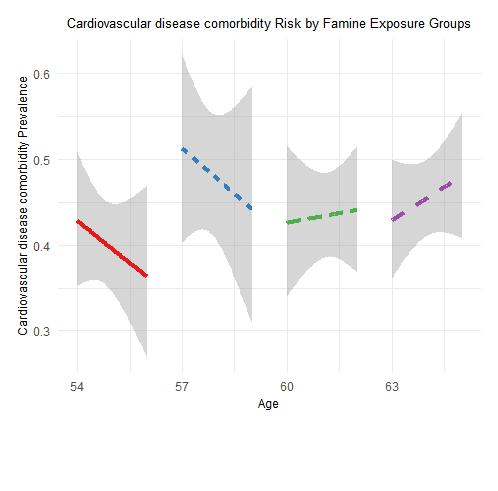 | 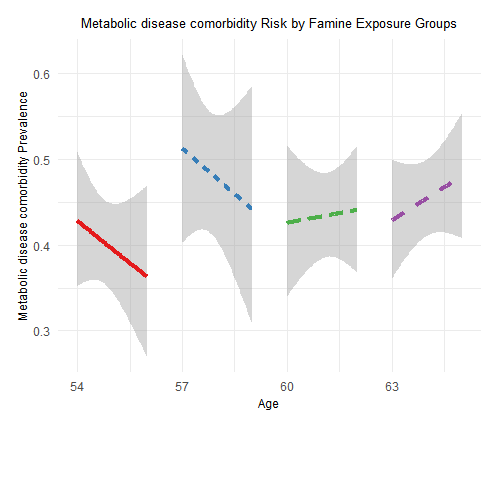 | 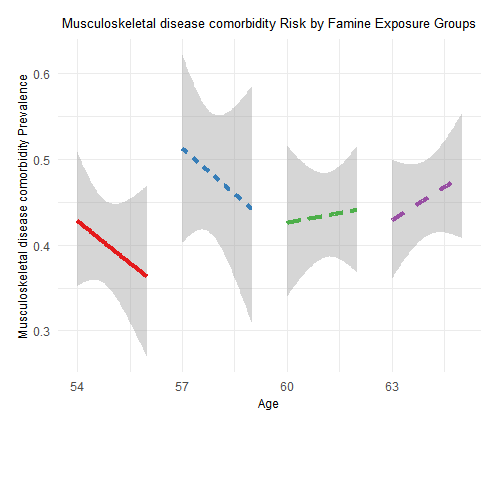 | 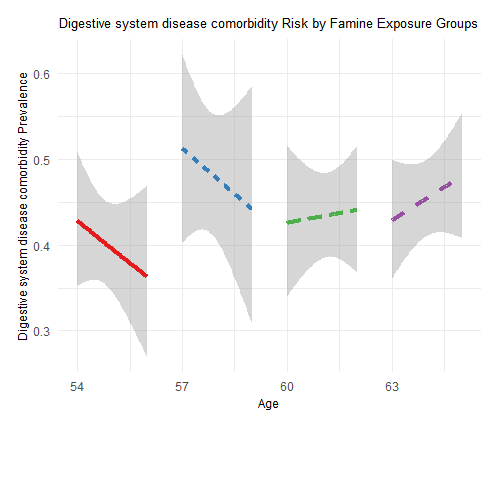 |
| --- | --- | --- | --- |
| 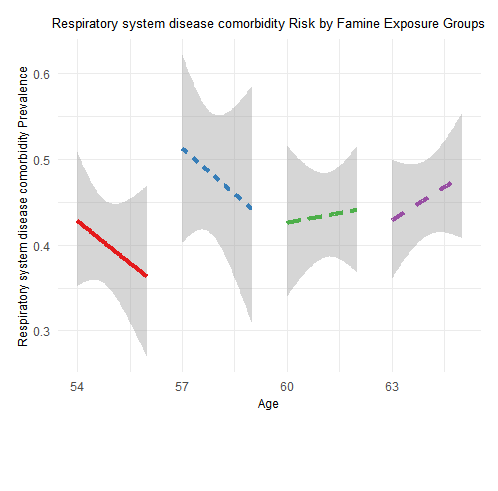 | 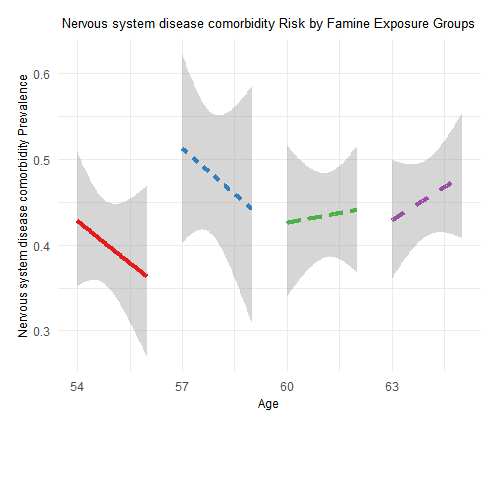 | 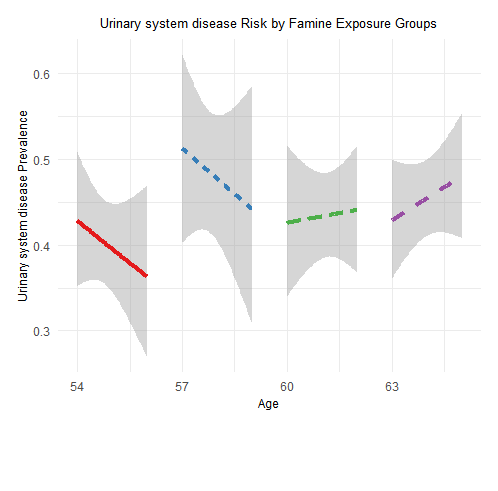 | 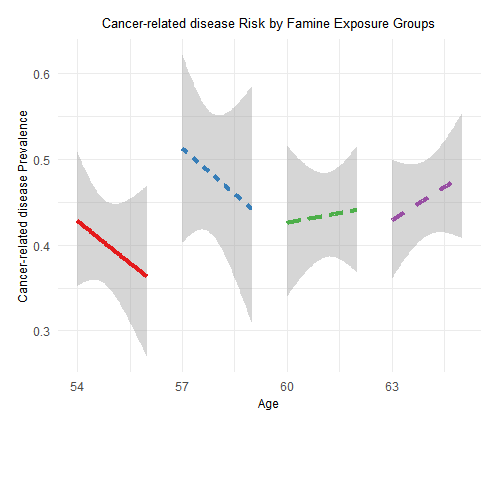 |

# **eFigure 3** Sensitivity Analysis on the Risk of Comorbidity between Famine Exposure and Chronic Diseases: using Age-Period-Cohort Analysis

# **Ethics approval**

Ethical approval for all the CHARLS waves was granted from the Institutional Review Board at Peking University. The IRB approval number for the main household survey, including anthropometrics, is IRB00001052-11015; the IRB approval number for biomarker collection, was IRB00001052-11014.

During the fieldwork, each respondent who agreed to participate in the survey was asked to sign two copies of the informed consent, and one copy was kept in the CHARLS office, which was also scanned and saved in PDF format. Four separate consents were obtained: one for the main fieldwork, one for the non-blood biomarkers and one for the taking of the blood samples, and another for storage of blood for future analyses.

# **Funding**

The National Natural Science Foundation of China (grant number 70773002, 70910107022, 71130002, and 71450001), Behavioral and Social Research division of the National Institute on Aging of the National Institutes of Health in the United States (grant numbers 1R21AG031372, 1R01AG037031, R03TW008358RO3AG049144, R01AG053228), the World Bank Group (contract number 7145915, 71592347172961), Chinese medical board (contract number 13-154, 16-249), and Peking University all provided critical financial support for our project.

**References**

1. Zeng Z, Bian Y, Cui Y, Yang D, Wang Y, Yu C. Physical activity dimensions and its association with risk of diabetes in middle and older aged Chinese people. *Int J Environ Res Public Health*. 2020;17(21):7803. doi:10.3390/ijerph17217803

2. Bai A, Tao L, Huang J, Tao J, Liu J. Effects of physical activity on cognitive function among patients with diabetes in China: A nationally longitudinal study. *BMC Public Health*. 2021;21(1):481. doi:10.1186/s12889-021-10537-x

3. FAN MY, LÜ J. Chinese guidelines for data processing and analysis concerning the International Physical Activity Questionnaire. *Chin J Epidemiol*. 2014;35((8)):961-964.
